# Preparation Strategy of Hydrogel Loaded with Natural Products and Its Research Progress in Skin Repair

**DOI:** 10.3390/gels12010062

**Published:** 2026-01-09

**Authors:** Lingchen Zhang, Qifan Li, Yuhan Zhou, Junran Yang, Xiaohang Sun, Xiaoyu Bi, Qiteng Ding, Xinglong Liu, Bo Yang

**Affiliations:** 1 College of Traditional Chinese Medicine, Jilin Agricultural Science and Technology University, Jilin 132101, China; m13569268135@163.com (L.Z.); zhouyuh221@163.com (Y.Z.); 2 College of Resources and Environment, Jilin Agricultural University, Changchun 130118, China; 18839892301@163.com (Q.L.); junran231201@163.com (J.Y.); 3 College of Chinese Medicinal Materials, Jilin Agricultural University, Changchun 130118, China; 18447375100@163.com (X.S.); bxy123163@163.com (X.B.); ding152778@163.com (Q.D.)

**Keywords:** natural product hydrogel, skin repair, tissue engineering, loading strategy, wound closure

## Abstract

Hydrogels are three-dimensional hydrophilic network structures with one or more polymers cross-linked, with excellent biocompatibility, drug-carrying function, and biodegradability. Meanwhile, skin wound repair includes hemostasis and coagulation, an inflammation stage, a proliferation stage, and a remodeling stage. Therefore, hydrogels loaded with natural products are widely used in repairing skin wounds through various mechanisms such as hemostasis, antibacterial activity, anti-inflammatory activity, angiogenesis promotion, skin regeneration, and skin repair monitoring. In addition, this study provides the cross-linking mechanism (physical cross-linking and chemical cross-linking) and construction mode (self-assembly and physical parcels) of the loaded natural product hydrogel. In general, the purpose of this paper is to comprehensively understand the mechanism and preparation strategy of hydrogels loaded with natural products for skin repair and provide theoretical reference for future skin repair research.

## 1. Introduction

As the first barrier between the human body and the external environment, the skin plays a vital role in protecting the human body. With the gradual progress of modern medical treatment, simple skin wounds have been well solved, but for serious skin wounds, simple medical treatment can no longer meet the needs of clinical treatment. From the perspective of wound healing time, skin wounds can be roughly divided into acute skin wounds and chronic skin wounds. Among them, acute wounds can be well healed, while chronic wounds usually do not spontaneously heal or even get worse because of the complexity of their treatment environment, and there is a certain chance of amputation or death. Skin repair is a dynamic and complex process of apoptosis and regeneration, roughly divided into four phases: coagulation and hemostasis, the inflammation phase, the proliferation phase, and the remodeling phase [1].

With the deepening of modern scientific research, people’s attention to health is gradually increasing. Traditional compounds have considerable therapeutic effects in skin repair but often have certain side effects or toxicity, which limits their use. However, natural compounds from plant extracts can effectively solve the side effects and toxicity problems in treatment and can effectively stimulate wound repair and promote the process of skin repair in treatment. Many previous studies have shown that natural products can be used at various stages of skin repair and are safer and more resistant than synthetic compounds.

In recent years, medicine has developed numerous dressings through the development of skin tissue engineering, e.g., gauze [2], films [3], foams [4], nanofiber membranes [5], hydrocolloids [6], and hydrogels [7]. Among them, hydrogel is composed of a three-dimensional network of hydrophilic polymers with physical or chemical cross-linking bonds, which can play a very good role in cell proliferation and adhesion. In addition, the special structure and high water content of hydrogel can absorb a large amount of cell exudate in skin repair and can also accelerate the massive diffusion of oxygen in cells to promote the speed of skin wound healing. On the other hand, hydrogels also have excellent swelling capacity, which can also absorb wound exudate, avoiding the risk of inflammation caused by possible secondary destruction.

**Figure 1:**
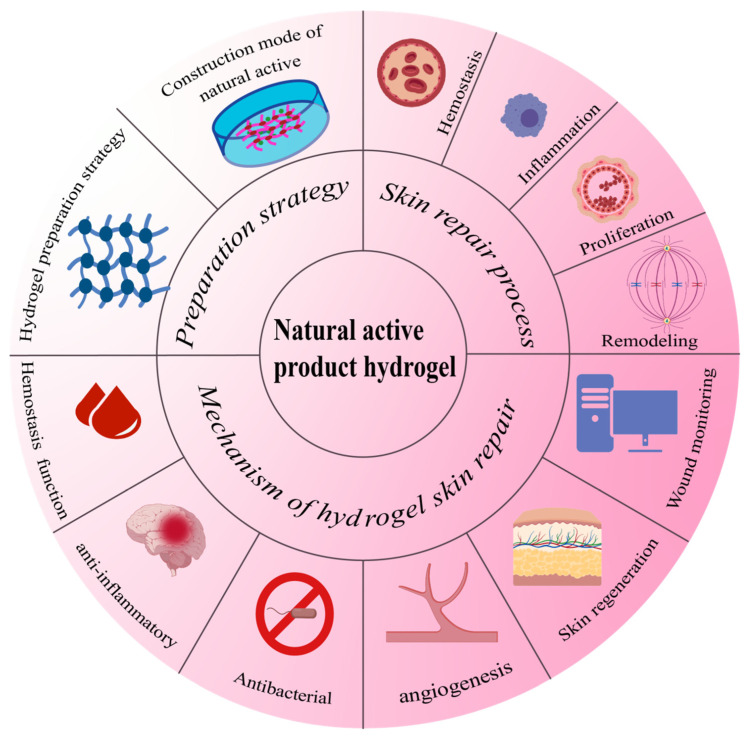
Natural active hydrogel skin repair process, repair mechanism, and preparation strategy.

Therefore, recent studies have attempted to enhance the medical properties of hydrogels by using them as carriers to carry a variety of ingredients with specific natural active ingredients [8]. This review focuses on the loading strategy of natural active product hydrogel, its role in skin repair, and the process of skin repair. The application of hydrogel loaded with natural active substances in skin repair is shown in Figure 1.

## 2. Skin Repair Process

**Figure 2:**
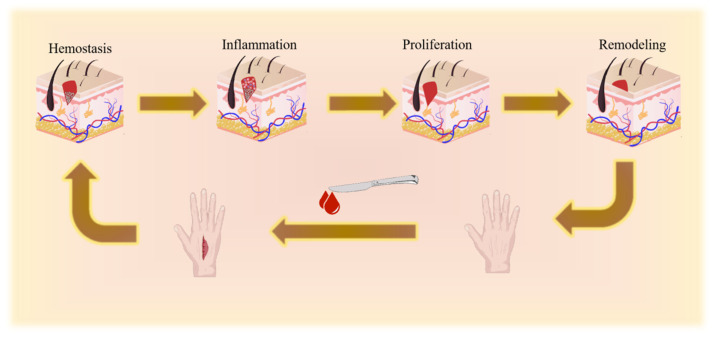
The process of skin repair includes hemostasis and coagulation, an inflammation stage, a proliferation stage, and a remodeling stage.

As the largest organ of the human body, the skin is often subjected to external environmental effects, resulting in a variety of injuries, such as acute skin damage and chronic skin damage [9]. The healing time for acute skin injuries is usually 2–4 weeks, and each stage is closely related during the repair process [10]. However, chronic skin injuries usually take a longer time to heal, often exceeding 4 weeks before fully healing and still being in the inflammatory and proliferative stages [11]. In some cases, acute injuries can turn into fatal or chronic injuries, and in most cases, even if the skin damage is repaired, it will still leave a scar. Therefore, this research on skin repair has been the focus of medical and medical aesthetic research for many years [12]. The skin repair process includes hemostasis and coagulation, the inflammation stage, the proliferation stage, and the remodeling stage, as shown in Figure 2 [13].

### 2.1. Hemostasis and Coagulation

**Figure 3:**
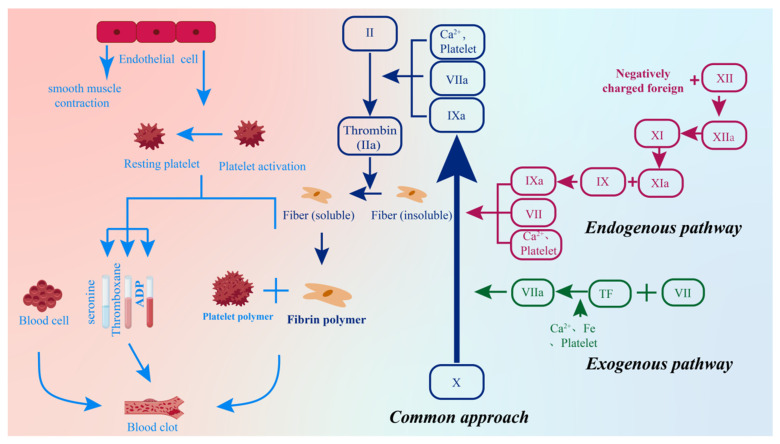
Thrombosis process (after vascular injury, the endogenous (XII-XI-IX) and exogenous (TF-VII) dual pathways converge at Xa, and thrombin is explosively generated on the surface of Ca^2+^ and activated platelets, rapidly converting fibrinogen into a cross-linked fibrin network and interweaving platelets, forming a stable thrombus).

After the skin is physically damaged by external factors, blood vessels rupture and bleed to flush out antigens and bacteria while triggering the first step in the skin repair process, hemostasis, to prevent excessive blood loss in the human body [1]. The process of hemostasis is physiologically manifested as the activation of platelet aggregation caused by the exposure of endothelial cells, collagen, and tissue factors, thereby releasing chemokines and growth factors to achieve hemostasis. Therefore, the hemostasis process can be roughly divided into two stages, primary coagulation (primary hemostasis) [14] and secondary coagulation (secondary hemostasis) [15], as shown in Figure 3.

Primary hemostasis is the first stage of the hemostasis process. Skin damage causes the smooth muscle of damaged blood vessels to contract and release signaling substances, reducing local blood loss and binding to platelet receptors during vascular contraction, promoting platelets to reach the site of injury, activating and releasing chemicals such as serotonin, thromboxane, and adenosine diphosphate, further enhancing vascular contraction, and ultimately forming platelet thrombi.

Secondary hemostasis is the process of forming fibrin coagulation on the site after primary hemostasis occurs. In this process, the activation of coagulation factors triggers the coagulation cascade and the production of thrombin, resulting in the conversion of plasma fibrin from soluble to insoluble and the formation of a biopolymer mesh of fibrin; eventually, together with the platelet thrombus, a thrombus is formed to immobilize the blood components. In this process, the coagulation cascade reaction, as the process of thrombospondin complex formation, can be divided into endogenous, exogenous, and common pathways [16].

Factor XII is activated into factor XIIa through contact with an external surface, which then activates factor XI to form factor XIa. Subsequently, with the participation of Ca^2+^ ions, factor XIa helps factor IX form factor IXa; finally, factor IXa combines with factor VIIIa to form the prothrombin complex. The exogenous pathway is caused by exposure to transmembrane glycoprotein tissue factor (TF) outside the bloodstream, mainly involving coagulation factors such as F, Ca^2+^, and factor VII. In the presence of Ca^2+^ ions, TF can bind to active coagulation factor VII to form the subunit VII tissue factor complex. The combination of endogenous and exogenous pathways is ultimately referred to as the common pathway, which involves the formation of prothrombin complex by Ca^2+^ ions Xa and Va factors and further activation into thrombin, promoting the conversion of fibrin and aggregation and completing blood coagulation through the synergistic action of fibrin, XIIIa factors, and blood cells [17]. This process aims to provide temporary treatment for skin wounds to prevent excessive pathogenic bacteria from entering the skin and disrupting the subsequent repair process.

### 2.2. Inflammatory Phase

One of the main stages for skin wound healing is the restoration of barrier function to prevent further injury or infection. Firstly, skin cells emit inflammatory signals, such as damage-related molecular patterns or pathogen-specific molecular patterns, which are recognized by Toll-like receptors at their site, triggering and maintaining inflammation [18]. Leukocytes, especially neutrophils, migrate with increasing gradients of chemokines until they reach the site of injury; during inflammation, neutrophils reach the site of injury and release chemokines such as interleukin 1-β (IL-1β), tumor necrosis factor-α (TNF-α), and interleukin 6 (IL-6) [19].

These chemokines amplify the immune response and attract monocytes for the ongoing healing process, which involves phagocytosis by neutrophils and monocytes. In addition, monocytes differentiate into macrophages that release transforming growth factors α and β (TGF-α and β) and insulin-like growth factor 1 (IGF-1), thereby facilitating the gradual transition from the inflammatory to the proliferative phase [20]. Activated regulatory T cells are part of the adaptive immune system. In addition to leukocytes, regulatory T cells are able to modulate tissue inflammation by attenuating interferon-gamma production and pro-inflammatory macrophage accumulation. According to relevant research, this action is mediated by the epidermal growth factor receptor pathway, which is used to promote skin wound repair [21].

The nature of the trauma may determine the length of the inflammatory phase. Acute skin wounds with large blood loss require longer hemostasis time, while chronic skin wounds that can be kept closed with simple pressure will coagulate faster [22]. Similarly, sharp injuries (e.g., incisions and laser wounds) will heal faster than wounds that produce necrotic tissue, whereas in chronic skin wounds, tissue regeneration stalls during the inflammatory phase, which can lead to pathological inflammation and failure to start wound healing at a late stage. After the inflammatory stage, the foreign cells and molecules in the skin repair part are fully removed, which clears the obstacles for the subsequent skin repair process.

### 2.3. Proliferative Phase

This process mainly occurs during days 4 to 14 of skin wound healing, which mainly involves epithelialization, neovascularization, and granulation tissue formation [13]. Epithelialization occurs during the early stages of skin repair, during which the original epithelial cells migrate upwards in a normal pattern if the basement membrane remains intact [23]. Epidermal progenitor cells are located underneath the repaired portion of the skin, and if the epidermal cells remain intact, the normal epidermal layer will return to its normal state within two to three days; if the basement membrane has been damaged, then the epithelial cells located at the edge of the skin begin to proliferate and send out protrusions to rebuild the protective barrier [24]. The angiogenesis stimulated by TNF-α is marked by the migration of endothelial cells and the formation of capillaries, and the migration of capillaries to the wound is crucial for the normal healing of the wound, as the granulation tissue formation period and tissue deposition require nutrients provided by capillaries. If the nutrients are insufficient, it can lead to long-term non-healing of the wound; subsequently, the epithelial cells located at the edge of the skin begin to proliferate and send out protrusions to re-establish a protective barrier against loss of body fluids and further bacterial invasion [25]. Epithelialization begins shortly after trauma and is first stimulated by inflammatory cytokines, with IL-1 and TNF-α upregulating keratinocyte growth factor (KGF) gene expression in fibroblasts; in turn, fibroblasts synthesize and secrete KGF-1, KGF-2, and IL-6, which stimulate neighboring keratin-forming cells to migrate, proliferate in the wound area, and differentiate in the epidermis [26].

The final part of the proliferative phase is the formation of granulation tissue, where fibroblasts migrate from surrounding tissues to the wound site, activate, and begin to synthesize collagen and proliferate. Platelet-derived growth factor (PDGF) is the main signal of fibroblasts, which comes from platelets and macrophages; the expression of PDGF in fibroblasts is amplified by autocrine and paracrine signal transduction [27]. Fibroblasts already located at the wound site will begin to synthesize collagen and transform into myofibroblasts for wound contraction, and compared to fibroblasts from the wound site, their proliferation is lower. In this process, new granulation tissue is formed, and the structural integrity of skin is rebuilt through epithelization and angiogenesis [28]. However, the regeneration of appendages such as hair follicles, sweat glands, and neurons is a challenge in adult wound healing, and further study on this stage may be very important to promote the study of skin repair.

### 2.4. Remodeling Stage

As angiogenesis is still occurring at the time of granulation tissue formation, this highly vascularized tissue presents clinically with a characteristic reddish coloration. Subsequently, it enters the final stage of skin repair, the remodeling stage, which mainly involves the remodeling of granulation tissue and the recombination of immature scars; this stage is also the most important stage in medical cosmetic treatment [29]. The main feature of the remodeling stage is the deposition of collagen into an organized and structured network. If there is matrix deposition at the site of skin repair, the difficulty of skin repair will be reduced, and excessive collagen synthesis will lead to hypertrophic scars or keloids [30].

This phase usually takes place two to three weeks after the start of skin repair, but the length of skin repair needs to be analyzed, with most repair processes lasting several months, and a few may last up to a year. The synthesis rate of collagen during skin repair comes not only from the increase in the number of fibroblasts but also from the net increase in collagen production in each cell. Fibroblasts begin to differentiate into muscle fibroblast contraction cells, characterized by high expression of alpha smooth muscle actin (α-SMA). MMP and other collagen types secreted by fibroblasts, macrophages, and other remaining cell types reshape the matrix, marking the formation of mature scars. The initially deposited collagen is thinner than uninjured skin, parallel to the skin. Over time, the initial collagen chains are reabsorbed, deposited thicker, and organized along the stress line [31]. These changes are also accompanied by an increase in the tensile strength of the wound, indicating a positive correlation between collagen fiber thickness, orientation, and tensile strength. As collagen recombination continues, cross-linking occurs between adjacent collagen bundles, leading to an increase in scar tensile strength. However, at the maximum value, the scar only recovers up to about 70% of its pre-injury mechanical strength [32]. Collagen found in granulation tissue is biochemically different from that of uninjured skin, with granulation tissue collagen having greater hydroxylation and glycosylation of lysine residues, and this increased glycosylation correlates with finer fiber size [33]. And researchers have shown that the collagen in scars, even after a year of maturation, is never as strong as the collagen in uninjured skin and that the wound strength never returns to 100 %, with wounds having only 3% of their final strength at 1 week; 30% at 3 weeks; and about 80% at 3 months [34].

Numerous researchers have shown that even after the remodeling phase, skin repair cannot fully restore the skin to its previously undamaged state. Faced with this problem, there is still a lot of room for skin repair. As the first physical therapy method that directly contacts the wound after disinfection, wound dressings should receive more attention and research from the medical, materials, and chemical communities. Research on wound excipients is also extremely important [35].

## 3. Function Mechanism of Loaded Natural Product Hydrogel for Wound Repair

**Figure 4:**
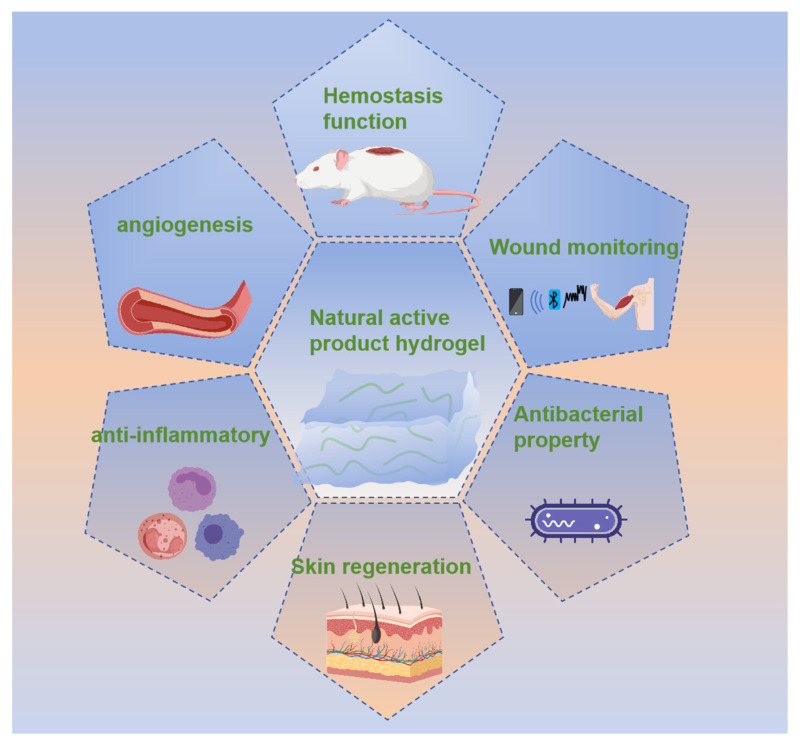
Mechanism of action of hydrogel for wound repair.

Skin, as the most frequently injured human organ in daily life, has a high incidence of undergoing the complex and diverse repair process involving a variety of factors, which often leads to poor healing, resulting in scars; this is also a difficult problem in medical field and tissue engineering in recent years [36]. Hydrogel loaded with natural products has unique advantages in repairing skin damage. Curcumin has been widely studied for its strong anti-inflammatory activity, but its extremely low water solubility and light instability put forward special requirements for the loading strategy. Therefore, researchers often use cyclodextrin inclusion or nano-lotion pre-dispersion to overcome this obstacle, but this increases the complexity of the system and may affect the homogeneity of gel. In contrast, water-soluble gallic acid is easier to load, but the hydrogel is required to have more accurate release response for better treatment. Whereas hydrogel is a potential tissue engineering material, the choice of polymers and changes in methodology have given hydrogel different properties to be applied at different stages of the skin repair process, as shown in Figure 4. The responsiveness, mechanical capacity, release characteristics, biocompatibility, safety, and functional differences of different hydrogels are summarized in Table 1.

### 3.1. Hemostatic Function

When the skin is subjected to physical or chemical impact, it may cause skin damage, and then the capillaries will rupture and blood will flow out. At this moment, it is very important to stop bleeding quickly. Hydrogels with remarkable hemostatic properties can significantly shorten and reduce the cell damage around the wound [45,46,47]. Generally, hemostasis involves three processes: vasoconstriction or blockage [48], formation of platelet blockage [49], and coagulation.

When bleeding occurs, blood vessels rupture and the body releases thromboxane and adrenaline, stimulating local and systemic vasoconstriction to reduce blood flow and loss [50]. Chitosan-based hemostatic hydrogels are often used for hemostasis due to their unique biocompatibility, tunable mechanical properties, injectability, ease of handling, and the ability of chitosan to aggregate blood cells and form embolisms to achieve hemostasis. Some researchers use the sol–gel method to synthesize nBG particles, which combine nano-bioglass (nBG) with silica, calcium, and phosphate ions on chitosan (Ch) hydrogel to achieve rapid coagulation. In the in vitro whole blood coagulation test and in vivo hemostasis evaluation, 2% Ch-5% nBG hydrogel stabilized a blood clot in 54 s in the case of liver bleeding and achieved hemostasis in 185 s in the case of femoral artery bleeding, which is more effective than 387 s with normal body mechanisms. Experiments have confirmed that the rapid and effective coagulation characteristics of 2% Ch-5% nBG hydrogel may be due to the synergistic effect of Ch and nBG in contact with blood [51].

In addition, the hydrogel dressing has a porous structure and rough surface, which can increase the contact area with blood, which is conducive to the rapid absorption of blood and accelerate hemostasis [52]. Shi et al. prepared a hemostatic hydrogel containing *L. barbarum* polysaccharide (LBP)-functionalized ultrathin MMT nanosheets (L-MMT NSs) for efficient hemostasis and wound healing, and the hydrogel showed a remarkably dispersed and porous 3D structure. They used block MMT in the hydrogel and successfully functionalized it with LBP-derived polysaccharides; in the hemostatic experiment of liver in vivo, compared with the control group, when P-L-MMT hydrogel was used, the blood loss was significantly reduced to 76 mg, which made the hydrogel show excellent hemostatic properties in the mouse model. The hydrogel prepared via this method significantly reduced the tissue damage caused by inflammation and significantly accelerated wound healing [53].

### 3.2. Antimicrobial Properties

If the skin is subjected to the proliferation and invasion of bacteria and microorganisms in the process of skin healing, it may lead to chronic skin wounds, delay wound healing, and even cause systemic infection in severe cases [54]. Therefore, hydrogel wound dressings should have excellent antimicrobial properties.

**Figure 5:**
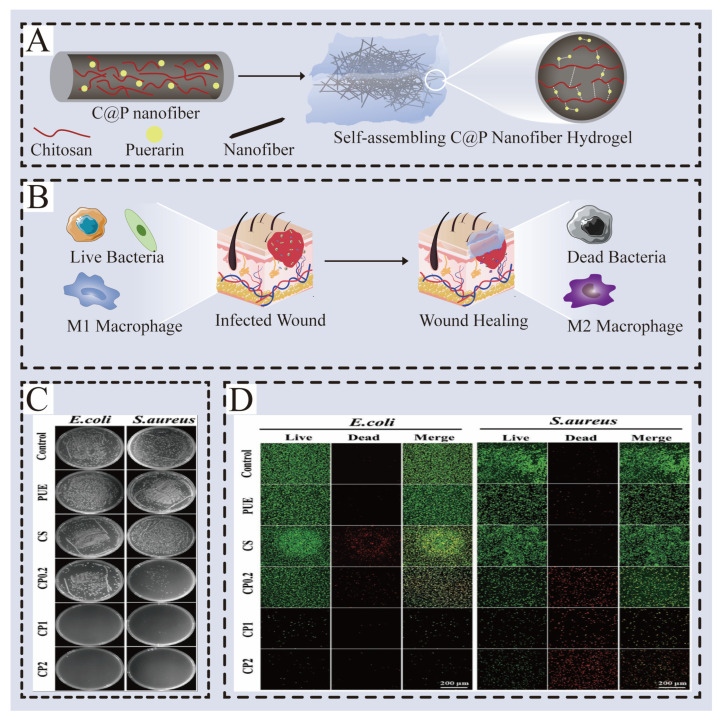
The mechanism of hydrogel to accelerate the healing of infected wounds by inhibiting bacterial growth and inducing macrophages to polarize into the M2 phenotype. (**A**) Schematic diagram of C@P hydrogel preparation process. (**B**) C@P hydrogel; bacteria in the infected wound could be rapidly killed, and macrophages were induced to polarize into the M2 phenotype to accelerate healing. (**C**) Photographs of bacterial colonies of *E. coli* and *S. aureus* from control, PUE, CS, CP0.2, CP1, and CP2 samples. (**D**) LIVE/DEAD staining of *E. coli* and *S. aureus* treated with control, PUE, CS, CP0.2, CP1, and CP2 samples. With permission from Wiley [55].

Pueraria lobata is a kind of Chinese herbal medicine with food and medicine homology. Puerarin extracted from Pueraria lobata has been found to form a kind of self-assembled hydrogel of Chinese herbal medicine under the mechanical action with chitosan through recent research; in SEM, it can be seen that the number of bacteria is obviously reduced. The antibacterial rate of hydrogel to *Escherichia coli* is 99%, *Escherichia coli* and *Salmonella* are 96% and 96%, respectively, and it can destroy the cell membrane of bacteria and inhibit the proliferation of bacteria, showing excellent antibacterial characteristics [55]. The mechanism of hydrogel to accelerate the healing of infected wounds by inhibiting bacterial growth and inducing macrophages to polarize into the M2 phenotype is shown in Figure 5.

Some hydrogels, such as chitosan and carboxymethyl chitosan, have certain antibacterial ability, but in the face of problems such as the aggravation of bacterial infection in the wound, the ideal antibacterial effect cannot be achieved only by the hydrogel material itself, so natural substances need to be added to the hydrogel to achieve the antibacterial purpose [56]. Gaona et al. prepared a chitosan hydrogel loaded with different concentrations of carvacrol, which was used as a medical material with good biocompatibility and antibacterial properties. In the bacteriostasis experiment, the bacteriostasis rate of the hydrogel against *Staphylococcus aureus* was ≥95%, and the bacteriostasis rate against *Escherichia coli* was ≤90%. In the drug release experiment, carvacrol can release drugs in chitosan hydrogel for 48 h continuously. In addition, the in vitro hemolysis experiment showed that the hemolysis rate of all hydrogels was less than 2%, and a hemolysis rate between 0 and 2% was considered non-hemolytic and safe for medical applications. This further confirmed that the hydrogel has certain reference value for the development of wound dressings [57].

### 3.3. Anti-Inflammatory Action

The inflammation stage is the second stage in the process of skin repair, and the regulation of inflammation on skin repair is extremely complicated. There is a risk of interruption of healing in both the early and late stages of inflammation, which is particularly obvious in chronic inflammatory wounds. In recent years, the ability of hydrogel wound dressings to mimic the function of the skin has received a great deal of attention in the field of scientific research. In view of the inflammatory stage of skin repair, we need to pay close attention to three aspects, namely the elimination of ROS (reactive oxygen species) [58], the isolation of chemokines [59], and the polarization of M1 to M2 of macrophages [60].

With regard to the removal of reactive oxygen species, Chen et al. studied a hydrogel for diabetes wounds to accelerate skin wound healing by removing ROS and other functions. In vivo results showed that H-SGPGA hydrogel significantly accelerated the process of diabetes wound repair (8.31 ± 5.54% of the wound surface on the 12th day) and promoted epidermal regeneration (79.13 ± 5.99%), collagen deposition (71.4 ± 9.1%), and angiogenesis (294.1 ± 29.6%). In vitro antioxidant testing showed that H-SG1GA (90.5 ± 0.4%) could play a synergistic antioxidant role between SGPGA and HXTL, and the ABTS clearance ability could reach 90.5 ± 0.45% within 2 h. In addition, H-SGPGA hydrogel also has an excellent DPPH clearance of 88.2 0.9%, showing excellent antioxidant performance [61].

Schirmer et al. designed a wound dressing scheme that can remove chemokines by using a validated biological hybrid poly (ethylene glycol)-glycosaminoglycan (starPEG-GAG) hydrogel. Based on the bionic reasonable design concept, this kind of hydrogel was integrated into the wound contact layer of composite fabric, and the efficient separation of CC and CXC family pro-inflammatory chemokines was achieved by regulating its local and overall charge density. In a mouse model of delayed healing, the dressing effectively alleviated the inflammatory response and promoted wound closure [62].

In the face of the above problems, the easiest way is to use hydrogel as a carrier and load natural active substances to enhance its anti-inflammatory activity. Zhou et al. developed a hyaluronic acid (HA) self-healing hydrogel (HA/SAB) containing salvinorin B (SAB) and investigated the anti-inflammatory mechanism of this hydrogel using q-PCR. They found that treatment with IL-4 significantly increased the expression of the M2 marker CD206, suggesting that it was successful in stimulating the M2 phenotype; in addition, SAB further increased the expression of CD206, suggesting a role for SAB in promoting M2 polarization and demonstrating the inhibitory effect of this hydrogel on the validation of M2 polarization by stimulating M1 in macrophages [63].

### 3.4. Angiogenesis

Angiogenesis is the growth of new blood vessels at the site of previously damaged blood vessels. As it has been proven that changes in their normal function can induce various diseases, the generation of new blood vessels has always been a focus of research for many scholars [64]. When the skin is damaged, inflammation stimulates blood vessels, and stimulated vascular endothelial cells will release matrix metalloproteinases (MMPs), thus decomposing the basement membrane. In this process, the activated endothelial cells will act as front-end cells, which will separate from the original position and move into the surrounding matrix, followed by proliferation of the endothelial cells to form new blood vessels [65].

**Figure 6:**
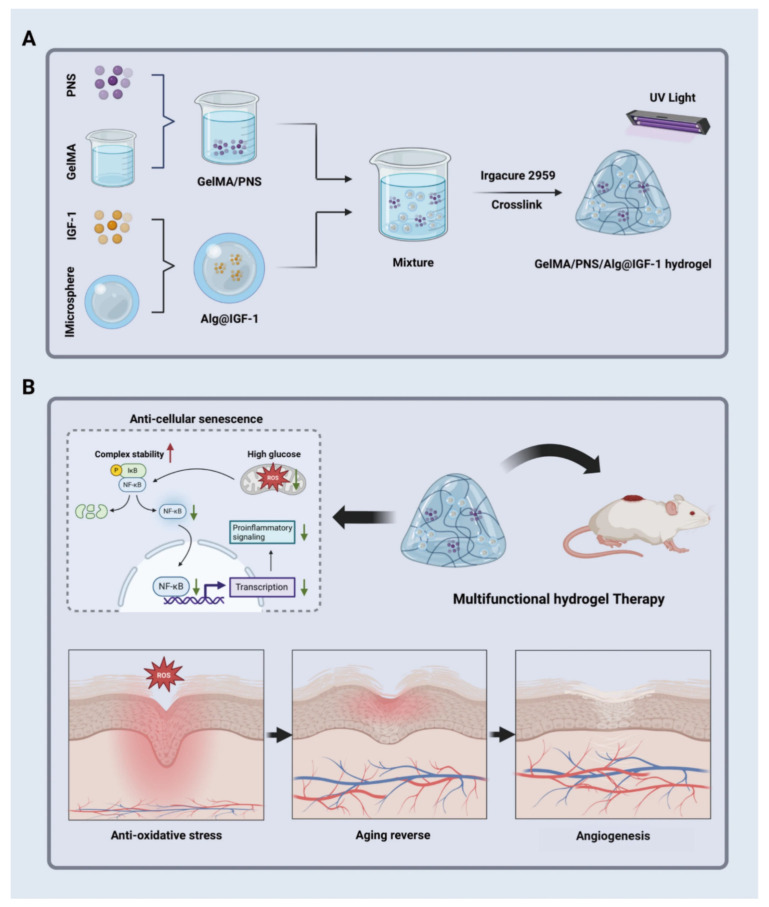
(**A**) Preparation of the multifunctional GelMA/PNS/Alg@IGF-1 hydrogel and (**B**) the intricate mechanism through which it facilitates diabetic wound healing; with permission from Springer Nature Link [66].

Hydrogel has become a promising skin healing dressing because it can create a suitable environment for skin repair, deliver drugs, and imitate the extracellular matrix. Yang et al. developed a diabetes wound-healing hydrogel based on glycyl methacrylate gelatin (GelMA), *Panax notoginseng* saponin (PNS), sodium alginate microspheres, and growth factor (IGF-1) and took the expression level of CD31 as the standard. CD31 staining showed that the neovascularization of the diabetes mouse model using hydrogel was significantly greater than that of the control group. The synergistic combination of PNS and IGF-1 established a favorable microenvironment for angiogenesis and promoted efficient and complete tissue repair [66]. The mechanism of hydrogel promoting angiogenesis and accelerating wound healing in diabetes is shown in Figure 6. In addition, the hyaluronic acid (HA) self-healing hydrogel (HA/SAB) of salvianolic acid B (SAB) explored by Zhou et al. found that the HA/SAB2.5 group displayed the highest expression of CD31 and α-SMA through immunofluorescence staining, showing excellent angiogenesis-promoting ability [63].

Xu et al., in their study on tissue engineering, found that magnetic hydrogels have special angiogenic ability and some magnetic guidance for tissue repair, and they developed a curcumin magnetic nanoparticle (CMNP) using a one-pot co-precipitation method and dispersed it in hyaluronic acid (HyA) to create an angiogenic magnetic hydrogel. In the research, it was found that the porous network of HyA composite hydrogels could be used as a matrix for sustained release of curcumin, thereby improving the angiogenic properties of the composite hydrogels [67].

### 3.5. Skin Regeneration

In the face of skin regeneration in the remodeling phase after the proliferation phase, how to restore the skin to its pre-breakage state has always been an important question in the medical community. Traditional wound dressings cannot absorb enough cell exudate and protect this part from microbial infection, which makes the skin regeneration occur as before [68]. Therefore, the development of hydrogel that can promote skin regeneration and restore the wound to the pre-injury quality is the main problem at present.

It is very important to study injectable hydrogel with high skin regeneration and healing characteristics for clinical application, Zhang et al. started with the high antioxidant properties of blueberry anthocyanin (BA) combined with carboxymethyl chitosan (CMCS) and oxidized hyaluronic acid (OHA) to explore the application of hydrogel in full skin regeneration and healing. In the wound healing experiment, 92.7% of the wounds in the experimental group were healed by the 12th day of the experiment, which significantly accelerated wound healing and promoted epithelial and tissue regeneration. In other studies, the composite hydrogel (Ag AV SF hydrogel) was prepared by introducing silver nanoparticles (AgNPs) and aloe vera (AV) as modifiers in the form of photocrosslinking, and a skin repair rat model was established for observation. It was found that the wounds of rats were resolved by wound contraction rather than re-epithelization, and the healing rate was significantly higher than that of other groups; meanwhile, the Ag-AV-SF hydrogel-treated group showed less scar tissue formation on days 7 and 21, which indicated that Ag-AV-SF hydrogel excelled in both promoting healing and inhibiting scar formation [69]. Hesperidin, as a bioflavonoid, has excellent efficacy in the treatment of wounds; however, its therapeutic use is limited due to poor water solubility and poor bioavailability. Praveen et al. attempted to develop hydrogel bandages with dendritic polymers to load hesperidin, and in vivo healing tests demonstrated that the 10% hesperidin-containing hydrogel exhibited the fastest wound closure (98.9% wound healing after 14 days) due to the properties of the hesperidin penetrating deep into the dermis with the help of the PAMAM dendritic macromolecule [70]. In addition, curcumin (Cur) can promote wound healing by affecting different stages of wound healing. Radha et al. prepared a physically cross-linked TOCN-PVA-Cur hydrogel with the addition of Cur via the freeze-thawing process and tested it in a rat model of skin repair, where H&E staining clearly showed that the epithelial cells of the experimental group were more completely repaired with this hydrogel than the control group [71].

### 3.6. Skin Repair Monitoring

For a long time, drug-resistant bacterial infections and biofilm formation have been challenges in biological protection, especially in the process of chronic skin repair. After the formation of skin wounds, monitoring the repair status and functional recovery during the repair process is essential. Therefore, it is necessary to have corresponding advanced materials to monitor the skin repair status at any time for disease assessment and subsequent treatment [72].

Based on the sensitivity of anthocyanins to pH changes, Zhao et al. synthesized light-curing hydrogels with EPLMA and blueberry anthocyanins (BAs) as precursors based on free radical polymerization reactions. The hydrogel is extremely sensitive to pH changes and can react quickly within 3 s, and the healing environment can be monitored by color change in response to pH changes, so using this characteristic to evaluate the change of wound pH environment plays an important role in the inflammatory stage and remodeling stage of skin repair and provides a potential treatment plan for related diseases [73]. In addition, inspired by anthocyanins, Zhang et al. performed extractions using mulberry fruits rich in anthocyanins and used their natural active substances to respond to pH and color changes. they cross-linked them with hyaluronic acid via the Schiff base reaction to prepare a “Tri Act” double-layer hydrogel dressing. The hydrogel not only has the characteristics of the natural active ingredients mentioned above but also has the ability to visually monitor infection, which simplifies the diagnosis process of skin repair. In addition, it shows therapeutic characteristics beyond conventional therapy in immunomodulation and promoting blood vessel regeneration and significantly enhances the therapeutic effect [74]. During the skin repair process, hydrogels are used as a way to maintain a moist repair environment for the skin, which promotes cellular activity and the re-epithelialization process, thus accelerating skin repair and reducing scar formation. In recent years, researchers have used turmeric extract (CLE) as a simple pH-sensitive indicator and loaded CLE into hydrogels made of hydroxyethyl cellulose grafted with green monomeric itaconic acid (IA) to prepare transparent, soft, and neutral pH-sensitive wound dressings [75]. Similarly, Ezati et al. also used curcumin in composite materials to achieve the purpose of pH response [76].

In addition, Hu et al. used the double-network hydrogel formed by covalent bonding and electrostatic interaction of chitosan and sodium alginate to load lignin Ag and quercetin–melanin (Q-Melanin) nanoparticles. Lig-Ag nanoparticles improve the conductivity and mechanization of hydrogels, which can be used for motion sensing and monitoring. Q-Melanin nanoparticles have anti-inflammatory and antioxidant effects, which, to some extent, enhance the anti-inflammatory properties of hydrogels and further improve the possibility of hydrogels acting on human monitoring, thus achieving accurate traction-assisted wound treatment [77].

Currently, many researchers focus on the degradation of hydrogels and drug release under various conditions (photodegradation, biodegradation, etc.), but there is a lack of research on the degradation of hydrogels in biological fluids, and it is impossible to determine the long-term release rate of drugs and the dynamic process of hydrogel degradation. Therefore, future research should carry out or simulate the removal rate of hydrogel in biological fluid so as to verify whether it matches the regeneration rate of wound tissue.

## 4. Application Strategies of Hydrogels with Different Cross-Linking Mechanisms to Promote Wound Repair

In recent years, due to its three-dimensional network structure, hydrogel has the advantage of versatility and applicability in the fields of biomedicine, environmental science, and materials engineering, a material with clear potential in science and industry. Hydrogels can be prepared in a variety of ways to dissolve natural products within the hydrogel for a wider range of uses, as shown in Table 2.

### 4.1. Preparation of Hydrogel

#### 4.1.1. Physical Cross-Linking

Physical cross-linking is mainly used to build hydrogels through ionic cross-linking [96], hydrophobic cross-linking, hydrogen bond cross-linking [97], crystalline cross-linking, and temperature-responsive cross-linking. Hydrogels constructed by physical action are usually less toxic or even non-toxic and may be more biocompatible. Physical cross-linking, also known as reversible cross-linking, is affected by changes in the pH, temperature and pressure in the external environment, which leads to the destruction or reconstruction of cross-linking points; however, due to the disadvantages of physical cross-linking, such as high production cost and weak mechanical strength of generated hydrogels, the application range of hydrogels prepared by physical cross-linking is limited [98].

##### Ionic Cross-Linking

Ionic cross-linking is the process of preparing hydrogels by ion exchange or electrostatic interactions. Two types of hydrogels can be made using this method: the first is a hydrogel produced by electrostatic interactions between a polyelectrolyte and oppositely charged multivalent ions and the second is a hydrogel produced by electrostatic interactions between two oppositely charged polyelectrolytes [99]. Compared with other methods, the hydrogel made by ionic cross-linking is more efficient and convenient.

Although traditional Ca^2+^-mediated alginate gels are famous for their rapid gelation, their long-term stability under physiological conditions is poor, which limits their effectiveness in medical applications. In a study using a novel catechol-modified sodium alginate (C-Alg) hydrogel system mixed with Ca^2+^ to initially form a hydrogel followed by enzyme-catalyzed gradual formation of chemical catechol cross-links, the C-Alg hydrogel showed significantly improved stability compared to the Ca^2+^-cross-linked hydrogel alone, with a gelation time of approximately 200 min without Ca^2+^, but was reduced to 20 min with increasing Ca^2+^ concentration [78]. Alginate brine gel is the most commonly used ionic cross-linked gel, which is cross-linked with divalent cations (such as Ca^2+^) using the internal gel technology. For example, calcium salts such as calcium carbonate are added into sodium alginate, and then Ca^2+^ is released through acidification such as acetic acid, which combines with the carboxyl group of alginate to form “egg box” gel. In addition, the stability and pore structure of the hydrogel can be controlled by adjusting the pH or ionic strength during the preparation process [100].

Liu et al. used sodium alginate (Alg), sialic acid (SA), arginine (Arg), strontium carbonate (SrCO_3_), and D-gluconate δ-lactone (GDL) to prepare hydrogels through ionic cross-linking for diabetes wound repair. On the seventh day, the repaired area reached 73%. Meanwhile, reducing inflammation by downregulating IL-6 and upregulating TGF-β expression and enhancing VEGF and bFGF expression promote angiogenesis, thereby accelerating wound healing [79].

##### Hydrophobic Cross-Linking

Hydrophobic cross-linking is a self-assembly process driven by non-covalent hydrophobic interactions, which can be prepared by mixing hydrophilic, hydrophobic, and surfactant monomers. The hydrogel prepared by hydrophobic cross-linking has the advantages of good toughness and fatigue resistance [101]. Common hydrophobic groups include alkyl groups, aromatic groups, etc.

Yang et al. prepared multifunctional injectable hydrogel dressing by using the hydrophobic effect of catechin’s benzene ring and the Schiff base reaction. The dressing has self-healing properties, with a wound closure strength of 63.4 KPa and a wound healing rate of 96.1 ± 0.9% after 14 days [80]. In addition, Ding et al. used carboxymethyl chitosan (CMCS) and oxidized dextran as carriers (Odex), loaded hydrophobic quercetin, and prepared injectable self-healing hydrogels through hydrophobic cross-linking. The release rate of the hydrogel reached 80% within 7 h, and the survival rate of cultured cells rose to more than 80% over time, showing good slow release and cell compatibility [81].

##### Hydrogen Bonding Cross-Linking

In physical cross-linking, hydrogen bonding cross-linking has become one of the most attractive directions for the development of self-healing polymers due to its dynamic nature, adjustable strength, and responsiveness to external stimuli [102]. Although the strength of a single hydrogen bond is not sufficient to induce supramolecular self-assembly behavior, when multiple hydrogen bonds are arranged to form a hydrogen bond array, both directionality and strength will be enhanced to some extent [103]. In addition, when the internal structure of the polymer provides a sufficient number of hydrogen bonding interactions, the composite can often be both self-healing and mechanically strong at the same time [104]. Therefore, the design of self-healing polymers with good hydrogen bonding ability has become a research hotspot in recent years.

Due to the spontaneity and reversibility of hydrogen bonds, the characteristics of hydrogel generated using hydrogen bonds will vary with temperature and pH [105]. In hydrogels made using non-covalent cross-linking techniques, hydrogen bonding is the most common method of preparation. However, the force of hydrogen bonding alone is very small, so it is often coupled with other cross-linking modes or multiple hydrogen bonds to produce gels.

Chen et al. used the helical structure of β-glucan extracted from Hericium erinaceus to promote its self-assembly through freeze-thawing and solvent exchange and introduced tannic acid to form injectable skin repair hydrogel with appropriate mechanical properties and biodegradability. The hydrogel can significantly enhance the expression of CD31 and promote angiogenesis, and the wound healing rate in 12 days in a mouse model is 96% [82]. In addition, in another study of physical and chemical double-cross-linked network hydrogel driven by hydrogen bonds, it is composed of natural product sodium alginate, N-[tris (hydroxymethyl) methyl] acrylamide, and polyethylene glycol diacrylate. The elongation of the gel can reach 700%, and the tensile elastic modulus is 27.7 KPa, which is helpful for wound closure. The cell compatibility test shows that the cell survival rate exceeds 90%, showing good biocompatibility and healing-promoting ability [83].

#### 4.1.2. Chemical Cross-Linking

Chemical cross-linking of hydrogels is usually characterized by structural stability, and in the cross-linking process, it is generally necessary to be assisted by cross-linking agents, which are mostly organic reagents; therefore, most chemical cross-linking presents a certain degree of toxicity, and the formation of hydrogels requires the removal of the cross-linking agent so that it does not participate in the reaction, with which it cannot achieve the purpose of direct use, which limits the application of chemical cross-linking [98]. Currently, the toxicity problem caused by the preparation of hydrogels by chemical cross-linking can be fundamentally solved by using non-toxic or low-toxicity cross-linking agents (natural polymer cross-linking agents). The introduction of dynamic covalent bonds such as imine bonds, borate bonds, and disulfide bonds aids the stability of chemical cross-linking and the reversibility/self-repair of physical hydrogel, and some dynamic bonds can be reversibly broken/formed in physiological environments, with better biocompatibility. In addition, optimizing the preparation process, improving the reaction efficiency and conversion rate, and reducing the use of toxic cross-linking agents is beneficial. Meanwhile, under high-temperature and weakly alkaline elution conditions, residual unreacted chemical cross-linking agents can be effectively removed, solving toxicity problems [106]. Chemical hydrogels are made by covalent cross-linking of polymers and exhibit a more durable and robust structure due to the presence of covalent bonds than physically cross-linked hydrogels, which rely on reversible interactions [107]. Chemical cross-linking usually includes radiation polymerization, free radical polymerization, click chemical reaction [108], enzyme cross-linking, and other methods.

##### Radical Polymerization

As the basic technology for industrial preparation of polymers and hydrogels, free radical polymerization forms a network structure through the free radical reaction of olefin monomers. Its process includes four steps—chain decomposition, initiation, propagation, and termination—and can build hydrogels containing specific functional groups [109]. Zhao et al. used the H_2_O_2_/horseradish peroxidase system to prepare hydrogels that can release drugs for a long time (up to 300 h, with a release rate of 80%) through the oxidative coupling of catechol groups and chemical cross-linking with amino groups. In a mouse model, the hemostasis time was significantly shortened by 268 s, and the wound was quickly closed (8.4 s) [84]. In addition, Bibire et al. polymerized sodium alginate and N-vinylcaprolactam to form thermosensitive hydrogel. After loading D-ketoprofen, they promoted drug targeted release through electrostatic repulsion at 37 °C, significantly accelerating skin repair. The wound closure effect after 30 h was better than that of the control group [85].

##### Radiation Polymerization

Radiation polymerization is the irradiation of a monomer or monomer solution by radiant light to produce primary reactive free radicals, which subsequently combine with other free radicals to form a hydrogel [110]. The hydrogels formed using this method do not require the intervention of cross-linking agents; this method is convenient, and the reaction conditions are easy to reach, but the monomer substances required are mostly toxic reagents, which presents some limitations for its application.

Demeter et al. prepared a hydrogel containing lavender oil through electron beam radiation polymerization (30 kGy). The gel has an ultra-high swelling rate (7700–18,000%) and controllable degradation (12% degradation in 35 days), can effectively absorb exudates, and plays a synergistic role as anti-inflammatory and moisturizing to promote skin repair [87]. Similarly, Mignon et al. used electron beam cross-linking of methacrylate-modified sodium alginate with diacrylate polyethylene glycol in an attempt to utilize the natural product sodium alginate with the compound’s own characteristics for the purpose of achieving high solubility and good moisturization with a transmittance rate of 80–95%, showing that it can be used as an effective photosensitizing agent carrier for applications in photodynamic therapy [88].

##### Enzymatic Cross-Linking

Enzymatic cross-linking is also known as a biological method, and in this modern world where safety is increasingly emphasized, this method is gradually demonstrating its research value and application potential [111]. Enzymatic cross-linking has the advantage of a fast and wide range of reactions without the intervention of toxic cross-linking agents but has the disadvantage of being influenced by a large number of factors, such as the concentration of the reagent, the concentration of the polymer, and the degree of polymer substitution. This method occurs mainly through the enzyme as a medium to promote the cross-linking of polymer materials, and as a result of the formation of a gel-like structure, enzyme cross-linking-prepared hydrogels are mainly used in cells [112], bioactive molecules, tissue engineering, drug delivery [113], and regenerative medicine in the drug.

Enzymatic cross-linked hydrogel shows good application potential in wound healing of diabetes. The biocompatible hydrogel designed by Wei et al. can regulate the expression of inflammatory factors. When its concentration is 40 mg/mL, the free radical clearance rate exceeds 96%, and the wound can be almost healed after two weeks of treatment [114]. In addition, a water gel was constructed through the enzymatic cross-linking of gallic acid and chitosan, which stabilized the antioxidant free radicals with the help of intramolecular hydrogen bonds, and achieved a free radical clearance rate of 90%, effectively promoting the proliferation and migration of fibroblasts under normal and hyperglycemic conditions, thus accelerating skin wound repair [88].

Although chemical cross-linking can produce a solid hydrogel network, its possible residual cross-linking agent toxicity limits its application in some tissue engineering applications. In contrast, although physical cross-linking has mild conditions and better biocompatibility, its mechanical stability and sensitivity to the environment (such as pH and ionic strength) are challenges that must be faced in practical applications. The dynamic covalent cross-linking that has emerged in recent years, such as the Schiff base reaction, provides a promising compromise solution for regulating biodegradability.

### 4.2. Construction Mode of Loaded Natural Active Hydrogel

#### 4.2.1. Self-Assembly

Self-assembly and physical cross-linking are both non-chemical cross-linking methods, and they do not involve the formation of chemical bonds but instead rely on the interaction between molecules to construct the network structure of hydrogels, which is promoted by the convergence of various non-covalent interactions [115], for example, hydrogen bonding, π–π stacking, hydrophobic interactions, van der Waals force, and electrostatic interactions. Self-assembled hydrogels are also affected by many factors, such as pH value, temperature, and so on. Self-assembly can be simply divided into single-molecule self-assembly and multi-molecule self-assembly.

##### Single-Molecule Self-Assembly

Single-molecule self-assembly is the spontaneous self-assembly of a single molecule through its own structural properties, such as functional group arrangement, hydrophobic interaction, etc. The spontaneous formation of an ordered network structure and further cross-linking to form a hydrogel, which has a high degree of spontaneity and biocompatibility, relies mainly on the intramolecular driving force to conduct [116]. Self-assembly of individual molecules to form hydrogels is mostly used to make specific materials, mainly for skin repair, drug delivery, and tissue engineering.

Gallic acid can self-assemble into hydrogel through π–π stacking and hydrogen bond interaction. Its FTIR spectrum shows that the intensity of the O-H stretching vibration peak weakens and moves to a low wave number after gel formation, indicating that cross-linking is mainly achieved through hydrogen bond interaction. In drug release, the release efficiency of gallic acid hydrogel at low concentrations can reach 95%, while the release efficiency of gallic acid hydrogel at high concentrations shows slow-release characteristics due to the denser network. The hydrogel can promote the basic repair of the epidermis after 9 days in the skin repair model [89].

Similarly, chlorogenic acid can also form hydrogel through π–π stacking and hydrogen bond self-assembly, and its release efficiency reaches 69% within 24 h, with good slow-release performance. In the rat skin repair experiment, the wound area of the hydrogel group was smaller than that of the control group on the 5th day, and it had basically healed on the 15th day, significantly better than other groups [90].

##### Multi-Molecular Self-Assembly

Multi-molecular self-assembly of hydrogels involves the polymerization of multiple molecules. A three-dimensional network structure is formed by intermolecular forces (such as electrostatic interaction, hydrogen bonding, van der Waals force, etc.). The network structure formed has regularity, stimulation response, self-healing ability, and dynamic reversibility, which makes them have potential applications in biomedical fields, especially in tissue engineering and wound dressing.

Yang et al. developed a dual-network cross-linked hydrogel composed of puerarin, silk fibroin, and gallium. The design aims to use the silk fibroin network to load puerarin to improve its solubility and enhance the mechanical strength of the gel. In a mouse model of liver hemorrhage, the amount of bleeding in the hydrogel group was only 13.3 mg, significantly lower than that in the blank group (113.3 mg). Its hemostatic mechanism was due to the physical barrier and the promotion of gallium ions in the endogenous coagulation pathway. After 21 days of the wound healing experiment, the hydrogel can effectively promote healing and minimize the scar area, which plays an important role in restoring skin function [91].

In addition, Wang et al. started from the chiral structure to overcome the difficulty of repairing skin infected with methicillin-resistant *Staphylococcus aureus* (MRSA) due to immune microenvironment imbalance and biofilm formation during the repair process. The hydrogel combines the two chiral substances with the betulinic acid alkaloids through self-assembly to synthesize the precursor substances, which were then combined to form a chiral hydrogel that showed excellent anti-hemolytic properties at an MIC of only 16 and showed significant efficacy 12 days after the use of the hydrogel, which further showed the potential application of natural small-molecule self-assembled hydrogels [117].

#### 4.2.2. Physical Parcels

Hydrogels made by this method have a wider range of applications, mostly for drugs that need to be released at a controlled rate, for protection, and for targeted release. The principle is that the drug is directly dispersed in the hydrogel precursor solution and is physically trapped in the physical pores of the hydrogel during the cross-linking process. There is no chemical bonding between the drug and the hydrogel for connection, which is mainly dependent on physical adsorption as well as spatial confinement.

Zhao et al. developed an injectable hydrogel to regulate the immune microenvironment and promote skin repair by using Astragalus polysaccharide, sodium alginate, and curcumin. The hydrogel was ultrasonically mixed in advance by physical embedding and then photo-cured so that curcumin was fully embedded in it. On the one hand, both of them provided an anti-inflammatory microenvironment. In the experimental verification, the fluorescence intensity of the hydrogel group was 8.37% lower than that of the LPS-induced inflammation control group, indicating that the hydrogel can transform macrophages from the M1 type to the M2 type, which has an anti-inflammatory effect. On the other hand, it promoted angiogenesis, and co-culture with human umbilical vein endothelial cells (HUVECs) promotes a 0.89-fold increase in the number of their nodes, confirming the potential of this hydrogel for angiogenic applications and realizing synergistic effects in the skin repair process [92].

Self-assembly and physical encapsulation are both hydrogel construction methods. In terms of construction mechanism, physical encapsulation forms a hydrogel network through physical interception and adsorption, which is mostly passive. Self-assembly spontaneously forms an ordered structure through non-covalent bonds. Structurally, self-assembled structures are relatively dynamic and reversible, whereas physical encapsulation is relatively static and relies on diffusion, gel swelling, or degradation in drug release. Both of them also have certain focuses in applications: self-assembly is more inclined to optimize the properties of the material itself, while physical encapsulation is more inclined to the loading and controlled release of the drug. In today’s and future developments, a combination of the two approaches is often used to balance material properties with therapeutic functionality, but much validation and experimentation are still needed to ensure safety and efficacy.

## 5. Conclusions and Prospects

Based on the extensive research and development of natural products in recent years, hydrogels have been studied more intensively in skin repair treatments. Due to the high water content, solubility, and special cross-linking structure of hydrogel materials, hydrogel medical materials are able to be used in different application scenarios for skin damage; for example, physical cross-linking, due to its non-chemical bonding, low toxicity, and high biocompatibility, is more suitable for the production of skin wound dressings, while chemical cross-linking is assisted by multiple chemical cross-linking agents, resulting in a more stable and robust architecture, and is commonly used in wound support scenarios [37]. In addition, the nature of hydrogel itself does not have a therapeutic effect, and the hydrogel loaded with natural active substances can fully expand the utilization range of hydrogels, effectively utilize the characteristics of hydrogel, and apply it to all stages of skin repair so that it is not limited to one stage, and the loading methods are self-assembly and physical wrapping, which can effectively load natural active substances [118].

However, with the improvement of the utilization rate of hydrogel loaded with natural active substances, there are also some problems that need to be seriously considered and excavated to determine the direction for improvement. Firstly, in terms of hydrogel performance, the mechanical strength, degradation rate, and activity release rate of hydrogel are still problematic; for example, natural polymer hydrogel is generally too low to be widely used in bone repair and other fields, and the rate of degradation is mostly affected by the environment and cannot match the rate of tissue regeneration. Meanwhile, many researchers focus on a few natural star products, and the exploration of many other potential compounds is insufficient. Moreover, studies on the loading and release of hydrogels loaded with natural products remain in vitro, and there is a lack of discussion on the release kinetics and degradation rate of hydrogels loaded with natural products in biological fluids or complex biological environments. In addition, in the process of applying hydrogel to clinical transformation, the consistency of hydrogel preparation and large-scale production, biological safety, and production cost control should be considered, which will restrict the development of hydrogel loaded with natural active substances. In terms of functional design, there is a lack of microenvironmental responsiveness, and hydrogels are mostly dependent on the influence of a single factor (e.g., pH, temperature, etc.), which makes it difficult to adapt to the dynamic multivariate changes in the skin wound healing process.

In the face of many existing problems, the optimization of hydrogel performance should focus on the balanced development of functional, clinical, and performance aspects, which can promote the development of standardized production processes and the construction of hydrogels to cope with the microenvironment of multiple intelligent responses. Although there is some progress in the loaded natural active substance hydrogel to cope with skin repair, there are still serious difficulties and challenges; further in-depth research needs to be conducted, and clinical trials to verify the loaded natural active hydrogel’s ability to cope with skin repair should be further undertaken.

## Figures and Tables

**Table 1:** Responsiveness, mechanical capacity, release characteristics, biocompatibility, safety, and functional differences of different hydrogels.

Name	Responsiveness	Mechanical	Release Characteristics	Biocompatibility and Safety	Function	References
PVA/CS/emodin hydrogel	pH responsive	The tensile strength is 1070 kPa, the fracture strain is 154%, the elastic modulus is 638 kPa, and the toughness is 803 kJ/m^3^, all of which are highest at the maximum concentration of emodin.	Rapid release	Low cytotoxicity; animal experiments showed no significant toxic reactions, but the high concentration of the emodin group (E_3_) inhibited cell growth and significantly decreased cell viability.	Promote cell proliferation and migration, enhance the expression of growth factors (EGF, VEGF-A, TGF-β 1, and bFGF), accelerate wound healing, promote angiogenesis and collagen deposition, resist bacteria and inflammation, maintain a moist environment of the wound, absorb exudates, and ensure that the hydrogel is intact and not easily damaged, reducing the risk of infection.	[37]
1&SAB hydrogel	ROS responsiveness	Rheological properties (G′ > G″); high elasticity (G′ and G″ remain stable in frequency scanning (0.1–100 rad/s)).	Rapid release	Low cytotoxicity; animal experiments have shown no significant toxic reactions.	Promoting cell migration (enhancing the migration ability of human dermal fibroblasts), antibacterial activity (effectively inhibiting *Escherichia coli*), antioxidant activity (effectively clearing ABTS free radicals and resisting oxidative damage induced by H_2_O_2_ at the cellular level), strong adhesion, promoting angiogenesis (increasing the number of CD31 positive blood vessels in wound tissue), promoting collagen deposition and tissue remodeling (accelerating collagen fiber deposition, promoting epithelialization, and reducing scar formation), and accelerating wound healing.	[38]
EGCG-NapFFY hydrogel			Continuous release (over 48 h)	No significant negative impact on overall animal health.	Extend the duration of drug action, promote wound healing, anti-inflammatory effect (can inhibit the release of pro-inflammatory cytokines), antioxidant, and antibacterial.	[39]
NPAC2	Temperature responsiveness		Continuous release (at least 7 days)	Hemolysis rate less than 2%, with good blood compatibility.	Promote wound healing (exerting anti-inflammatory, antioxidant, collagen synthesis, and tissue remodeling effects, accelerating the healing process).	[40]
HA-GB			Continuous release		Promote wound healing of diabetes, anti-inflammatory (reduce the level of pro-inflammatory cytokines (TNF-α, IL-1 β, and IL-6) in the wound, inhibit the NF-κB signal pathway), promote epithelization (increase the expression of TGF-β in the wound and improve epidermal hyperplasia), promote angiogenesis (increase the expression of VEGF in the wound and promote the formation of new blood vessels), and regulate collagen remodeling (promote collagen deposition and increase the ratio of type I collagen to type III collagen, which is conducive to tissue maturation).	[41]
BCC		Shear thinning characteristic, injectable; BCC hydrogel undergoes gel–sol transition when the strain is 35%.		The hemolysis rate is below 5%, and the cytotoxicity is low.	Promote wound healing of diabetes, resist bacteria, promote the proliferation and migration of L929 fibroblasts, downregulate the expression of pro-inflammatory factor TNF-α in wound tissue, upregulate the expression of anti-inflammatory factor IL-10, inhibit the expression of the NF-κB signaling pathway (p65) and iNOS, promote angiogenesis (increase the expression of vascular endothelial growth factor (VEGF-A) and the number of new CD31-positive blood vessels in wound tissue), and regulate the polarization of macrophages (promote the polarization of macrophages from pro-inflammatory M1 to repair-promoting M2).	[42]
NC@Gel	Temperature responsiveness	Shear thinning behavior (viscosity decreases with the increase of shear rate); gel strength (G′ > G″) forms stable solid hydrogel.		Hemolytic activity below 4%, with no significant cytotoxicity; animal experiments show no significant toxic reactions.	Accelerate wound healing of diabetes, resist oxidation (effectively eliminate ROS in cells and mitochondria, increase the activity of antioxidant enzymes such as SOD and CAT, and reduce the level of MDA), resist inflammation (inhibit NF-κ B signal pathway, reduce the secretion of pro-inflammatory factors such as TNF-α, promote the polarization of macrophages from pro-inflammatory M1 (CD86^+^) to anti-inflammatory M2 (CD206^+^), and increase the level of IL-10), regulate mitochondrial function (reduce the production of mitochondrial ROS), and promote tissue repair (promote angiogenesis, promote collagen deposition and tissue remodeling, and promote cell proliferation and migration).	[43]
HG_MTx/HG_CMx	Temperature responsiveness	Hardness HG_MTx (9.7 N), adhesion HG_MTx (20.3 kPa) and HG_CMx (21.3 kPa); rheological properties (G′ > G″) form stable solid hydrogel.	Continuous release (96 h)	No significant cytotoxicity.	Antibacterial (*Staphylococcus aureus* and *Escherichia coli*), antioxidant, anti-inflammatory, and promotes wound healing.	[44]

**Table 2:** Summary of names of some hydrogels, natural substances contained, cross-linking modes, types of applications, and mechanisms of action.

Names	Natural Substances Contained	Cross-Linking Modes	Types of Applications	Mechanism of Action	Reference
C-Alg	Catechol and sodium alginate	Ionic cross-linking/enzymatic cross-linking	Drug delivery	Dual synergistic mechanism of ionic cross-linking and laccase-catalyzed enzymatic chemical cross-linking	[78]
ASASG	Sodium alginate	Ionic cross-linking/hydrogen bonding/electrostatic interactions	Antioxidant, hemostatic, and diabetic wound healing	Multi-target synergistic action of ‘antioxidant, anti-inflammatory, pro-angiogenic, and collagen remodeling’	[79]
U-COC	Catechin	Hydrophobic cross-linking/π–π stacking/hydrogen bonding	Antioxidant, antibacterial, and skin repair	Multi-target synergistic mechanism of ‘antioxidant, antibacterial, anti-inflammatory, and tissue regeneration promoting’	[80]
CMCS/Odex/Que-PF127	Quercetin	Hydrophobic cross-linking/physical embedding	Antioxidant, antibacterial, and skin repair	Composite system of ‘CMCS-Odex dynamic network + quercetin–PF127 micelle’	[81]
HEBG/TA	Monkey head mushroom extract	Hydrogen bonding	Antioxidant, antibacterial, and skin repair	Synergistic effect of ‘physical cross-linking network + biologically active ingredients’	[82]
THMA/PEGDA/SA	Sodium alginate	Hydrogen bonding/free radical polymerization	Skin repair	Mechanism of ‘chemical–physical dual network + hydrogen bond dynamic response’	[83]
PCPD/AS@APF/H_2_O_2_	Catechol	Free radical polymerization	Antibacterial and scar repair	Mechanism of ‘rapid gel–mechanical adaptation–antibacterial and anti-inflammatory–promoting regeneration’	[84]
DEXHY	Sodium alginate	Free radical polymerization/physical embedding	Skin repair	Mechanisms of ‘temperature-sensitive carriers–controlled drug release–microenvironmental regulation’	[85]
LO	Lavender oil	Radiation polymerization	Anti-inflammatory and skin repair	Synergistic mechanism of ‘highly absorbent porous structure + slow release of bioactive ingredients + pH-responsive degradation’	[86]
AlgMA/PEGDA	Sodium alginate	Radiation polymerization/free radical polymerization	Wound repair	Mechanism of ‘covalent crosslinking initiated by electron beam + natural–synthetic polymer complementarity’	[87]
CGA	Gallic acid	Enzyme cross-linking	Antioxidation	Mechanism of ‘enzymatic cross-linking–antioxidant–cell regulation’	[88]
GA	Gallic acid	Hydrogen bonding/π–π stacking	Antibacterial, anti-inflammatory, and skin repair	Mechanism of ‘self-assembled fiber network–dynamic mechanical properties–slow release of biological activity’	[89]
CA	Chlorogenic acid	Hydrogen bonding/π–π stacking	Anti-inflammatory and skin repair	Triple mechanism of ‘self-assembled fiber network–dynamic mechanical adaptation–inflammation/angiogenesis coupling regulation’	[90]
Puerarin-SF-Ga	Puerarin	Hydrogen bonding/π–π stacking	Antibacterial, anti-inflammatory, antioxidant, and skin repair	Triple mechanism of ‘double nanofiber network–ion synergistic antibacterial–biological activity–slow release’	[91]
OCS/NX@Cur	Sodium alginate and curcumin	Free radical polymerization/physical embedding	Anti-inflammatory and antioxidant	Synergistic mechanism of ‘double-network mechanical support–sustained release of supramolecular drugs–immune microenvironment regulation’	[92]
rGO/BGs@PDA-loaded CS-HEC/AgNCs hydrogel (bioactive glass)	Chitosan, hydroxyethyl cellulose, and dopamine	π–π stacking/hydrogen bonding/free radical polymerization	Antibacterial and skin repair	Multi-mode synergistic mechanism of near-infrared and photothermal silver ion spatiotemporal synergistic antibacterial, biologically active ion mediated tissue regeneration, and conductive antioxidant microenvironment regulation	[93]
FABA (bioactive glass)		Dynamic covalent cross-linking (Schiff base reaction)	Antibacterial and skin repair	Multifunctional synergistic mechanism of Cu^2+^/alendronate sodium synergistic antibacterial + downregulation of TNF-α/upregulation of IL-4/IL-10 anti-inflammatory + promotion of re-epithelialization and skin appendage regeneration	[94]
CaP NPs (calcium phosphate)	Methyl cellulose	Hydrophobic cross-linking	Repair bone defects	Synergistic bone regeneration mechanism of “thermal responsive gel network + in situ formation of bioactive nanoparticles”	[95]

## Data Availability

The data presented in this study are openly available in the article.
